# A New Human NHERF1 Mutation Decreases Renal Phosphate Transporter NPT2a Expression by a PTH-Independent Mechanism

**DOI:** 10.1371/journal.pone.0034764

**Published:** 2012-04-10

**Authors:** Marie Courbebaisse, Christine Leroy, Naziha Bakouh, Christine Salaün, Laurent Beck, Bernard Grandchamp, Gabrielle Planelles, Randy A. Hall, Gérard Friedlander, Dominique Prié

**Affiliations:** 1 Faculté de Médecine, Université Paris Descartes, Paris, France; 2 Research Center, Growth and Signaling, INSERM U845, Paris, France; 3 Service de Physiologie - Explorations Fonctionnelles, Hôpital Necker-Enfants Malades, Paris, France; 4 Hôpital Bichat Claude Bernard, Institut Fédératif de Recherche 02, INSERM, Université Paris Diderot, Paris, France; 5 Department of Pharmacology, School of Medicine, Emory University, Atlanta, Georgia, United States of America; 6 Service de Physiologie - Explorations Fonctionnelles, Hôpital Européen Georges Pompidou, Paris, France; University of Pittsburgh, School of Medicine, United States of America

## Abstract

**Background:**

The sodium-hydrogen exchanger regulatory factor 1 (NHERF1) binds to the main renal phosphate transporter NPT2a and to the parathyroid hormone (PTH) receptor. We have recently identified mutations in NHERF1 that decrease renal phosphate reabsorption by increasing PTH-induced cAMP production in the renal proximal tubule.

**Methods:**

We compared relevant parameters of phosphate homeostasis in a patient with a previously undescribed mutation in NHERF1 and in control subjects. We expressed the mutant NHERF1 protein in Xenopus Oocytes and in cultured cells to study its effects on phosphate transport and PTH-induced cAMP production.

**Results:**

We identified in a patient with inappropriate renal phosphate reabsorption a previously unidentified mutation (E68A) located in the PDZ1 domain of NHERF1.We report the consequences of this mutation on NHERF1 function. E68A mutation did not modify cAMP production in the patient. PTH-induced cAMP synthesis and PKC activity were not altered by E68A mutation in renal cells in culture. In contrast to wild-type NHERF1, expression of the E68A mutant in *Xenopus* oocytes and in human cells failed to increase phosphate transport. Pull down experiments showed that E68A mutant did not interact with NPT2a, which robustly interacted with wild type NHERF1 and previously identified mutants. Biotinylation studies revealed that E68A mutant was unable to increase cell surface expression of NPT2a.

**Conclusions:**

Our results indicate that the PDZ1 domain is critical for NHERF1- NPT2a interaction in humans and for the control of NPT2a expression at the plasma membrane. Thus we have identified a new mechanism of renal phosphate loss and shown that different mutations in NHERF1 can alter renal phosphate reabsorption via distinct mechanisms.

## Introduction

The maintenance of normal serum phosphate concentration is critical for many biological processes including bone mineralization and cell function. The kidney plays a pivotal role in phosphate homeostasis. It adapts urinary phosphate excretion to balance the quantity of phosphate absorbed by intestine or stored by cells and bone. Phosphate is filtered at the glomerulus and mainly reabsorbed in the renal proximal tubule through the type 2a sodium-phosphate cotransporter (NPT2a) and to a lesser extent through the type 2c sodium-phosphate cotransporter (NPT2c) [1] . The magnitude of renal phosphate reabsorption relies on the level of expression of NPT2a at the apical plasma membrane of proximal tubular cells. Defects in NPT2a activity, as observed in patients with loss-of-function mutations of NPT2a or in mice with NPT2a gene ablation, results in hypophosphatemia with reduced capacity of the kidney to reabsorb phosphate (TmP/GFR) and increases the risk of renal lithiasis or bone demineralization [2], [3], [4], [5]. Two hormones reduce NPT2a expression at the plasma membrane: parathyroid hormone (PTH) and fibroblast growth factor 23 (FGF23). PTH removes NPT2a from the apical plasma membrane by stimulating cyclic adenosine monophosphate (cAMP) production through PTH type 1 receptor (PTH1R). The amount of cAMP produced in response to PTH in proximal tubular cells is modulated by the sodium-hydrogen exchanger regulatory factor 1 (NHERF1) [6], [7].

NHERF1 is a protein with two structural domains named PDZ1 and PDZ2. These domains bind to the carboxy-terminal parts of transporters or receptors. Hence, NHERF1 binds to the c-terminal extremity of PTH1R and decrease PTH-induced cAMP synthesis [6], [7], [8]. We have recently identified mutations of NHERF1 in patients with hypophosphatemia, low TmP/GFR and normal serum PTH and FGF23 concentration [9]. The mutations were located in the PDZ2 domain of NHERF1 or in the inter-region domain. Contrary to wild type NHERF1 protein, the mutant NHERF1 proteins were unable to control PTH-induced cAMP production in renal cells in culture.

Co-immunoprecipitation experiments showed that NHERF1 also binds to the c-terminal part of NPT2a. This binding may preferentially involve the PDZ1 domain of NHERF1 [10], [11]. Several data suggest that NHERF1-NPT2a interaction is mandatory to maintain a proper expression of NPT2a at the apical domain of proximal tubular cells. Hence, disruption of NHERF1 gene in mice decreased NPT2a expression in renal brush border membranes [12]. However the complete invalidation of NHERF1 expression abrogates not only the NPT2a-NHERF1 interaction but also the association of NHERF1-PTH1R. Manipulations of NHERF1 that exclusively alter NPT2a-NHERF1 association are lacking to support the physiological importance of this interaction. We report here the characterization of a naturally occurring NHERF1 mutation identified in a patient with a decreased capacity of the renal tubule to reabsorb phosphate. We show that this mutation impinges renal phosphate transport by a mechanism distinct from that reported for previously identified NHERF1 mutants.

## Methods

### Objectives

We described the consequences of a previously unidentified mutation in the PDZ1 domain of NHRF1 gene. We analyzed the mechanism by which this mutation can impair renal phosphate reabsorption.

### Participants and ethics

The patients provided written informed consents to obtain blood DNA samples for genomic DNA extraction in accordance with French national guidelines. This study was approved by the local ethic comitee: Comité Consultatif de Protection des Personnes en Recherches Biomédicales Bichat Claude Bernard. Sequencing was performed on genomic DNA obtained from patients who were referred to our clinical department for renal assessment before initiating potentially nephrotoxic treatment for psoriasis, or before heminephrectomy for living kidney donation, or for examination of renal lithiasis or bone demineralization. In these subjects we measured serum phosphate and PTH concentrations and calculated the TmP/GFR value using Walton and Bijvoet nomogram [13].

### Sequencing

Both strands of the coding region and the intron-exon junctions of NHERF1 were sequenced by investigators who were unaware of the clinical history and the biological parameters of the patients. The sequences of the intronic primers used for the polymerase chain reaction have already been published [9]. The PCR products were sequenced with the use of the BigDye Terminator Cycle Sequencing Ready Reaction kit (Perkin Elmer on an ABI PRISM 3100 sequencer (Applied Biosystems). Both strands were analyzed using the software program Sequencing Analysis (Applied Biosystems) and Clustal W (Infobiogen). The nucleotide position was numbered according to the starting point of the ATG codon in the complementary DNA (cDNA) (sequence accession number NM_004252).

Biochemical measurements of electrolyte and hormone concentration were based on standard clinical laboratory procedures. Plasma FGF23 concentration was measured with an Elisa assay (Human FGF-23 (C-Term) ELISA Kit, Immutopics, Inc, San Clemente, CA, USA).

### Cell culture

Opossum kidney (OK) cells and Hela cells were obtained from the American Type Culture Collection. OK cells were cultured in DMEM/Ham's F12 containing sodium bicarbonate, 14 mM; Hepes, 20 mM pH 7.40; sodium selenite, 50 nM; L-glutamine, 2 mM; Insulin, 5 µg/ml; 5 mg/ml transferrine; Hydrocortisone; defined bovine serum, 2.5% under a 5% CO2-containing atmosphere.

HeLa cells were cultured in DMEM with glucose, 4.5 g/l; Glutamine, 200 mM; Sodium Pyruvate, 1 mM; Hepes 20 mM and defined foetal bovine serum, 5% under a 5% CO2-containing atmosphere.

### Plasmid construction

Wild type NHERF1 cDNA was inserted into pEF1-B plasmid by PCR amplification, and subcloned into pEF1-B (Invitrogen) plasmid using EcorV-Not1 enzymes. NHERF1 mutants were generated from the wild type cDNA with the use of Quick Change Site-directed Mutagenesis kit (Stratagene). All constructs were verified by sequencing. Flag or GFP tags were inserted at the N-extremity of the protein.

#### Cell transfections

Subconfluent cells were transfected with the lipofectamine reagent and 0.5 µg/ml of NHERF1 vectors or 0.25 µg/ml of human SLC34A1 plasmid according to manufacturer's instructions (Invitrogen).

Expression in Xenopus laevis oocytes, Voltage-Clamp experiments. Oocytes were surgically harvested from Xenopus laevis obtained from Centre de Recherche de Biochimie Moléculaire (CNRS Montpellier, France) with the agreement of the Comité Régional d'Ethique pour l'Expérimentation Animale Ile de France Paris Descartes. Wild type or mutant NPT2a or NHERF1 cDNAs were introduced in the RNA expression vector pSP64T. RNA was synthesized with the use of the Riboprobe in Vitro Transcription System kit (Promega). Defolliculated oocytes were injected with 10 ng of wild type NPT2a (SLC34A1) RNA and 24 hours later with 10 ng of NHERF1 RNA. Phosphate-induced current was measured as previously described with an imposed holding potential of −50 mV [4].

#### Phosphate uptake experiments

Radiolabeled phosphate uptake was measured by incubating cells for 10 min in isoosmotic transport medium containing or not Na and 0,5 mCi/ml ^32^P. Cells were washed 3 times with cold medium (pH 7,4) and solubilized with 0,5% triton X-100. The radioactivity of the lysate was counted by liquid scintillation spectroscopy. Each assay was performed in triplicate.

Intracellular cAMP content was measured in OK cells pre-incubated for 30 min at 37°C, under a 5% CO2-containing atmosphere in culture medium containing 3-isobutyl-1-methyxanthine, 1 mM and exposed afterwards to 10^−7^ M PTH, diluent (negative control) for 5 min at 37°C, under a 5% CO2-containing atmosphere. Total intracellular cAMP quantification was performed using Cyclic AMP Competitive ELISA Kit (Thermo, France) according to manufacturer's instructions.

PKC activity was determined using the Promega SignaTECT PKC Assat system, which contains a specific PKC substrate (biotinylated Neurogranin_28–43_). This ^32^P-labeled substrate was recovered with a streptavidin matrix according to the manufacturer's instructions (Promega Corp. WI, USA).

Biotinylation and isolation of cell surface proteins were performed in HeLa cells transfected with pEF1-B empty plasmid, NHERF1 wt or E68A mutant and NPT2a-peGFP, using Cell Surface Protein Isolation Kit (Pierce) according to the manufacturer's instructions.

Antibodies used. HRP-conjugated anti Flag M2 antibody was purchased from Sigma chemical co. Anti NHERF1 antibody was purchased from Santa Cruz biotechnology. Anti GFP antibody was purchased from Chemicon (Millipore). Anti Na-K-ATPase was purchased from Abcam.

### Statistical analysis

statistical tests used are indicated where appropriate.

## Results

### Case report

**Figure 1:**
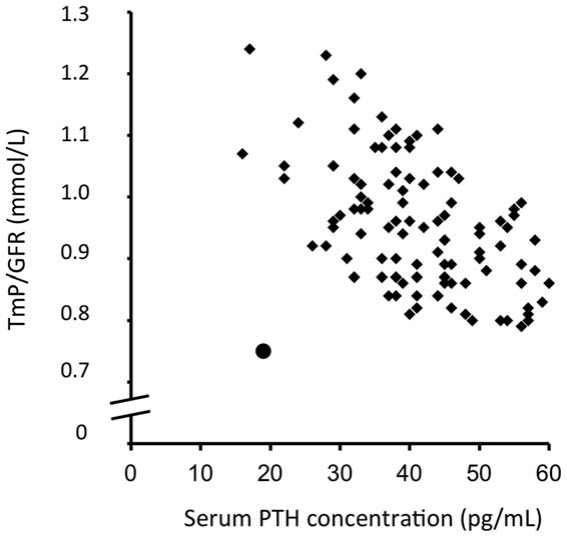
Relationship between serum PTH concentration and the capacity of kidney to reabsorb phosphate normalized for the glomerular filtration rate (TmP/GFR) in 112 control subjects (diamonds). The patient with the mutation in NHERF1 gene is represented by a circle.

The mutation was identified in a 71 year-old patient who was referred to our clinical department for renal calcium lithiasis and serum ionized calcium concentration in the upper normal values, associated with hypercalciuria (Table 1). Serum phosphate concentration and TmP/GFR were below the lower limit of the normal values contrasting with serum PTH concentration within the low normal range (Table 1). The study of the relationship between TmP/GFR and serum PTH values in a group of 112 control subjects clearly showed that TmP/GFR was uncommonly low in face of serum PTH concentration in this patient (Figure 1). This difference was not explained by serum FGF23 concentration, which was in the low range (Table1). Urinary cyclic AMP excretion was normal. Serum FGF23 concentration was not increased. Plasma concentration of 1,25 (OH)_2_ vitamin D was slightly elevated. Thyroid hormone measurement discarded hyperthyroidism. Bone mineralization was normal at the lumbar spine (T-score −0.9) and slightly decreased at the radius (T-score −1.4) and at the hips (T-score −1.6). The patient exhibited no other sign of proximal tubule dysfunction: glycosuria, aminoaciduria was absent and serum bicarbonate concentration was normal. The diagnosis of impaired renal phosphate reabsorption was retained. We sequenced both strands of the genes encoding the renal sodium-phosphate co-transporters SLC34A1 (NPT2a) and SLC34A2 (NPT2c) and found no mutation.

**Table 1:** Biochemical data of the patient.

	Patient	Normal Range
Serum Phosphate (mmol/l)	0.83	0.85–1.40
TmP/GFR (mmol/l)	0.75	0.75–1.40
Serum ionized calcium (mmol/l)	1.28	1.15–1.30
Serum PTH (pg/ml)	19	11–60
Serum FGF23 (RU/ml)	18	<120
Serum 1,25-dihydroxyvitamin D (pg/ml)	48	16–42
GFR (ml/min)	100	>70
Urine Calcium/creatinine (mmol/mmol)	0.51	<0.48
Urinary cyclic AMP excretion (pmol/min)	2700	3200±600

**Figure 2:**
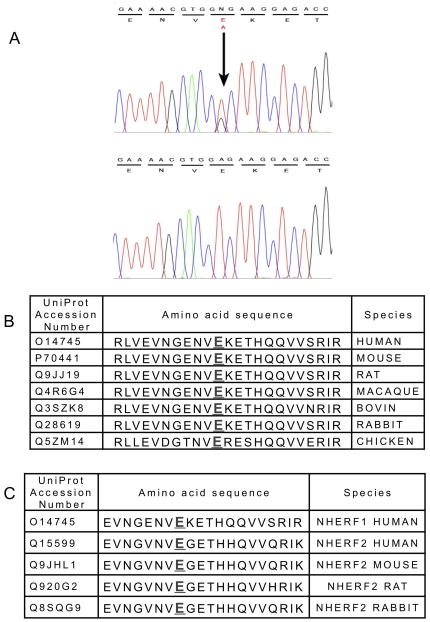
Localization of the mutation. A: Sequence analysis of genomic DNA from the patient with NHERF1 mutation. The upper panel shows the portion of the DNA sequence from the patient with NHERF1 mutation. The sequence can be compared with that obtained in a subject with no mutation (lower panel). The nucleotide and the amino acid sequences (above and below the horizontal line respectively) are indicated. A vertical arrow points the mutation. The patient was heterozygous for the mutation. Conservation of the NHERF1 protein sequence surrounding the mutation site in various species. The amino acid modified by the mutation is highlighted. B: Alignment of human NHERF1 amino acid sequence around the site of mutation with homologous sequences of NHERF1 in various species. C: Alignment of human NHERF1 amino acid sequence around the site of mutation with homologous sequences of NHERF2 in various species.

We sequenced the coding region and the intron-exon junctions of the NHERF1 gene and identified a previously unreported mutation. The patient had a c.203 A → C heterozygous transition in exon 1, which substituted alanine for glutamic acid at position 68 (p.E68A) (figure 2 A). The alignment of the NHERF1 protein sequence showed that glutamic acid 68 was highly conserved in the NHERF1 PDZ1 domain throughout evolution and in the PDZ1 domain of NHERF2 of various species (figure 2 B, C) suggesting that it plays an important role in the function of this domain. Furthermore this variant was not found in the 1000 genomes database (http://browser.1000genomes.org).

The patient had no relatives alive, consequently we could not study the segregation of the mutation with a low TmP/GFR. However to determine if this mutation could be present in subjects with normal renal reabsorption of phosphate we sequenced NHERF1 in a population of 112 subjects with normal TmP/GFR and we did not find a single example of the E68A mutation.

**Figure 3:**
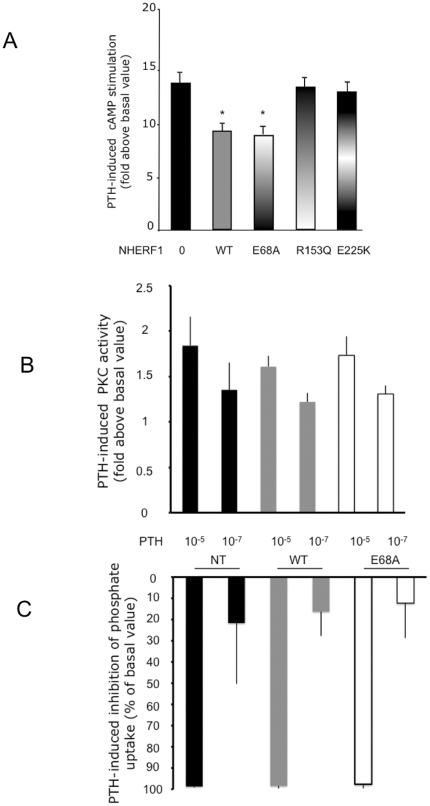
Effects of NHERF1 mutations on cAMP production, PKC activity and phosphate uptake in response to PTH stimulation. cAMP accumulation, PKC activity and phosphate uptake inhibition by PTH were measured in opossum kidney (OK) cells transfected with a plasmid alone or containing either the wild type cDNA of NHERF1 or cDNA from mutant NHERF1. The mutant E68A has been identified in the patient reported in the present study, the mutants R153Q and E225K have been characterized in a previous study (9). A: Cells were stimulated with PTH 10^−8^ M. We present the increase in cAMP synthesis above the values measured in the absence of PTH. Results are means ± SD of four independent experiments. The groups were compared with the use of Kruskall-Wallis test (overall P = 0.001, * p<0.05 compared to condition in the absence of NHERF1). B: PKC activity in cells stimulated with 10^−5^ M or 10^−7^ M of PTH. For each PTH concentration used, PTH induced PKC activity did not differ in OK cells expressing wild type NHERF1 or the E68A mutant. Results are means ± SD of three independent experiments. C: The inhibitory effect of PTH on phosphate uptake was determined at two concentrations of PTH (10^−5^ M or 10^−7^ M). For each PTH concentration, the magnitude of the PTH-induced inhibition on phosphate uptake did not differ between OK cells expressing wild type or E68A NHERF1. Results are means ± SD of three independent experiments.

We have previously reported, in patients with impaired renal phosphate reabsorption, mutations in the NHERF1 protein that induced an increase in PTH-induced cAMP production [9]. We evaluated cAMP production in response to PTH in OK cells, a model of renal proximal tubular cells. OK cells were transfected with a control plasmid or with a plasmid containing either the wild type cDNA, or the mutant E68A, or previously identified mutants (R153Q or E225K). PTH markedly stimulated cAMP synthesis in OK cells expressing the control plasmid. As previously observed, cAMP production was blunted in cells expressing wild type NHERF1 cDNA and this effect was not observed in the presence of R153Q or E225K mutants. By contrast cAMP stimulation was similarly decreased in cells transfected with the E68A mutant or the wild type NHERF1 cDNA (Figure 3A). Since PTH receptor can also signals through PKC pathway we also compared PTH-stimulated PKC activity in OK cells transfected with empty plasmid or with wild type NHERF1 or the E68A mutant (Figure 3B). OK cells were stimulated with two concentration of PTH. PTH-induced PKC activity was similar in all conditions (Figure 3B). To make sure that the E68A mutant did not interfere with another PTH-induced signaling pathway that could inhibit phosphate uptake we compared the inhibitory effect of PTH at two concentrations in OK cells expressing wild type NHERF1 or the E68A mutant (Figure 3C). As expected PTH inhibited phosphate transport at both concentrations with a similar magnitude under all experimental conditions.

**Figure 4:**
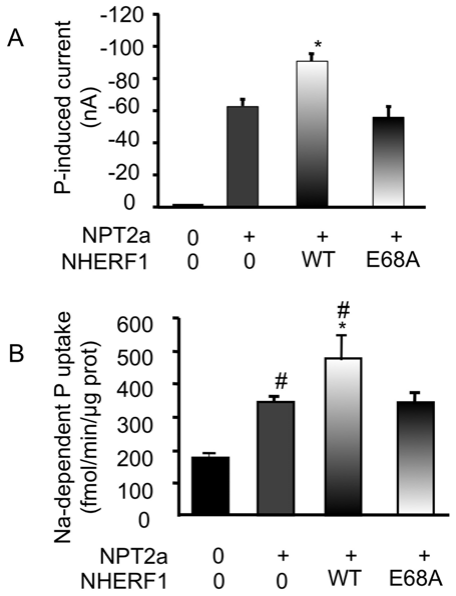
Measurement of phosphate transport in cells expressing the wild type or the E68A mutant NHERF1 together with the sodium phosphate cotransporter NPT2a. Panel A: Phosphate-induced current recorded in Xenopus oocytes injected with water or with either cRNA of NPT2a alone or in association with the cRNA of the wild type (WT) NHERF1 or the E68A mutant. Results are means ± SD, n = 9. Overall comparison was performed with the use of the Kruskall-Wallis test (P<0.0001) then the group expressing NPT2a alone was compared with other groups with the use of the Mann-Whitney test (p<0.01). Panel B: Sodium-dependent phosphate uptake was measured in Hela cells transfected with empty plasmids or with the plasmid containing the cDNA of NPT2a alone or together with the cDNA of the wild type or the NHERF1 mutant. Results are means ± SD, n = 6. Overall comparison was performed with the use of the Kruskall-Wallis test (P<0.0001) then the group expressing NPT2a alone was compared with the other groups with the use of the Mann-Whitney test (* p<0.01 vs NPT2a+WT NHERF1, # p <0.001 vs NPT2a alone).

We then asked whether E68A mutant might modify phosphate uptake by the main renal phosphate transporter NPT2a (SLC34A1). Wild type or E68A cDNA were co-expressed with NPT2a in *Xenopus* oocytes and phosphate uptake was assessed by the measurement of sodium-dependent phosphate-induced current. Expression of NPT2a alone markedly increased phosphate-induced current (figure 4A). Co-expression of NPT2a with wild type NHERF1 further increased phosphate uptake. By contrast the magnitude of phosphate transport in *Xenopus* oocytes expressing the E68A mutant and NPT2a was similar to that measured in the absence of NHERF1 (figure 4A). We reproduced these experiments in cultured human epithelial cells (Hela cells) in culture that do not express endogenous NPT2a or NHERF1 (see supplementary data). Expression of NPT2a alone in these cells significantly raised sodium-dependent phosphate uptake above basal values (figure 4B). Expression of wild type NHERF1 produced an additional increase in sodium-dependent phosphate transport. However phosphate uptake was similar in Hela cells expressing NPT2a alone or together with E68A NHERF1 mutant.

**Figure 5:**
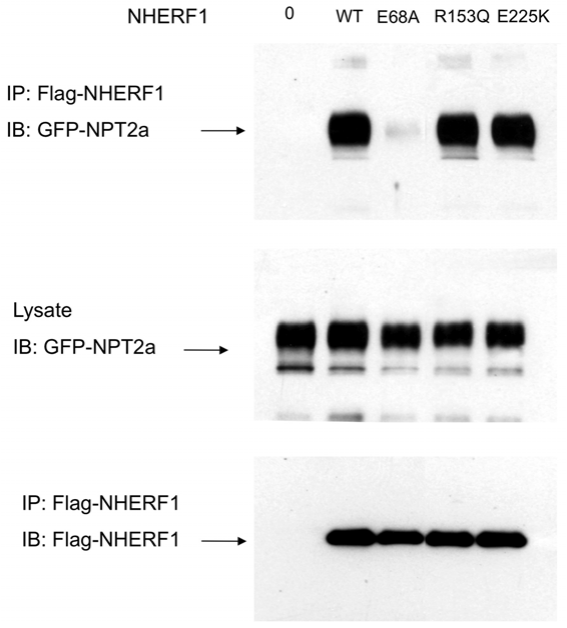
Co-immunoprecipitation of NHERF1 with NPT2a in Hela cells. Hela cells were transfected with a plasmid containing the cDNA of NPT2a tagged with GFP at its N- extremity. Cells were also transfected with a plasmid containing the wild type cDNA of NHERF1 or one mutant (E68A or R153Q or E225K) tagged with Flag. Upper panel: Flag-tagged-NHERF1 protein was immunoprecipitated by using an anti-Flag antibody and the immunoprecipitates were probed with anti-GFP antibodies to detect GFP-tagged-NPT2a on Western blot. Middle panel: The total expression of GFP-tagged-NPT2a was analyzed in the cell lysates by anti-GFP Western Blot. Lower panel: Wild type and mutant Flag-NHERF1 was immunoprecipitated and probed by using anti-Flag antibodies to compare Flag-NHERF1 immunoprecipitation in the different conditions.

To determine whether the E68A mutation impinged on the NHERF1-NPT2a interaction we performed co-immunoprecipitation experiments in cells expressing tagged NPT2a and wild-type or mutated NHERF1 proteins. The amount of wild type and mutated NHERF1 proteins and of NPT2a protein did not differ between the various experimental conditions (figure 5 middle and lower panels). Immunoprecipitation of wild-type or mutant R153Q or E225K NHERF1 proteins co-immunoprecipitated similar amounts of NPT2a protein. By contrast, immunoprecipitation of the E68A protein co-immunoprecipitated only faint levels of NPT2a protein (figure 5 upper panel).

**Figure 6:**
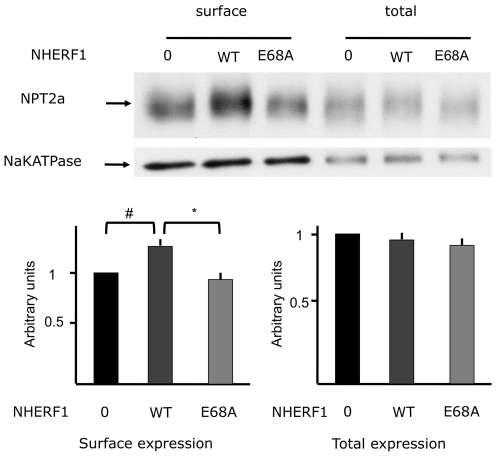
Effect of mutant NHERF1 on the cell surface expression of NPT2a assessed by biotinylation experiments. Hela cells were transfected with GFP-tagged-NPT2a alone or together with a plasmid containing the cDNA of the wild type NHERF1 or the E68A mutant. NPT2a proteins labelled with sulfo-NHS-SS-biotin were revealed by using anti-GFP antibodies. Labeled proteins were isolated with avidin-agarose beads according to manufacturer's manual (Pierce). Proteins eluted from the beads and total cell lysate were loaded on a SDS gel. Western blot was probed with anti-GFP antibodies. Na+ K+ APTase was used as a loading control and detected with specific antibodies. The higher panel shows a western blot representative of four experiments. Lower panel: Statistical analyses of the quantification of the western blot (n = 4). Results are presented as mean ±sd. Quantification of the surface expression of NPT2a: Anova p = 0.038. Post Hoc test: Tukey-Kramer multiple comparisons test (#: p<0.05; * p<0.01).

We assessed the amount of NPT2a protein present at the plasma membrane by biotinylation experiments in cells transfected with NPT2a cDNA alone or with a plasmid containing wild-type or mutant NHERF1 cDNA. The total amount of NPT2a protein was unchanged by the expression of wild type or E68A mutant NHERF1 (figure 6). However NPT2a protein abundance at the cell surface differed between the experimental conditions. Expression of the wild type NHERF1 was associated with a significant increase in NPT2a targeting to the plasma membrane compared to cells expressing NPT2a in the absence of NHERF1 (figure 6). Conversely the expression of the E68A mutant was associated with a decrease in NPT2a levels at the cell surface (figure 6).

## Discussion

We report here the functional characterization of a previously unknown mutation in the NHERF1 gene identified in a woman who had renal lithiasis and a decreased capacity of kidney to reabsorb phosphate contrasting with serum PTH concentration in the low normal range. Since the patient had no children and no relatives alive, we were not able to study the segregation of the mutation with the phenotype of renal phosphate leak. However the role of the mutation in the phenotype of the patient is supported by several lines of evidence: the absence of this mutation in 224 alleles of subjects with normal TmP/GFR, the uncommon relationship between serum PTH concentration and TmP/GFR value, the conservation of the amino acid throughout the evolution, and, above all, the functional consequences of this mutation on the expression and activity of the major renal sodium-phosphate cotransporter NPT2a when expressed in epithelial cells and in oocytes. The mutated amino acid is strongly conserved in the PDZ1 domain of both NHERF1 and NHERF2 of various species suggesting that it plays a pivotal role in the function of this domain. The E68A mutation differs from those previously identified by its localization in the PDZ1 domain and by the mechanism thorough which it decreases NPT2a expression at the cell surface. The previously reported mutations of NHERF1were located in the PDZ2 domain or in the region between PDZ1 and PDZ2 domains. We have shown that these mutations modify NPT2a expression only in the presence of PTH, by increasing cAMP production by the PTH1R and that urinary cAMP excretion was increased in the patients exhibiting these mutations [9]. In contrast, urinary cAMP excretion was normal in the patient with the E68A mutation and the mutated protein retained the capacity of decreasing PTH-induced cAMP production similarly to wild-type NHERF1. These results suggest that the PDZ1 domain is not crucial for the interaction between NHERF1 and PTH1R in human. By contrast, our data show that the PDZ1 domain is critical for the interaction between NHERF1 and NPT2a. We found that the expression of the wild-type NHERF1 in human epithelial cells that lack NHERF1 augmented NPT2a location at the plasma membrane. These results are in line with previous reports showing that re-expressing NHERF1 in renal proximal tubular cells of NHERF1 null mice increased NPT2a expression [14]. At variance with wild-type NHERF1, the E68A mutant failed to increase NPT2a expression at the surface of human cultured cells and, consequently, did not enhance phosphate uptake. This lack of effect of the E68A mutant was likely due to a defect in the interaction between NPT2a and E68A NHERF1 as suggested by the lack of co-immunoprecipitation of NPT2a with the E68A mutant. In the present study we did not determine if this interaction was direct or indirect. Experiments performed in yeast [10] suggested that the C-terminal part of NPT2a interacts predominantly with the first PDZ domain of NHERF1. Pull down experiments performed in OK cells overexpressing NHERF1-PDZ1 showed an interaction between PDZ1 and NPT2a [15]. However it has not been reported that a specific disruption of PDZ1 or a mutation in this domain could alter NPT2a targeting to support the physiological role of PDZ1. Using a non-natural occurring mutation Weinman demonstrated that phosphorylation of ser 77 of NHERF1 was mandatory to release NHERF1 NPT2a interaction, which allows NPT2a internalization [16]. We report a naturally occurring mutation of a highly conserved amino acid in PDZ1 that prevents NPT2-NHERF1 binding. The PDZ2 domain might plausibly compensate for an alteration of PDZ1. However our data show that this defect cannot be compensated by the presence of PDZ2. These findings demonstrate that NHERF1 PDZ1 has a central role in the functional interaction between NHERF1 and NPT2a and in controlling phosphate homeostasis in humans.

In the present case the data observed in a patient are consistent with those obtained in a knockout animal model. Indeed the phenotype of the patient with the E68A mutation, which associates kidney stones, renal phosphate wasting, a rise in calcitriol concentration, increased urinary calcium excretion, low PTH levels and the absence of modification of PTH signaling by the mutant, is very similar to that observed in NHERF1 knock-out mice [12].

The patient presented with a defect of renal phosphate transport suggesting that in human renal proximal tubular cells, other proteins with PDZ domains such as NHERF2 cannot compensate for the defect of NHERF1. In the rat, NHERF2 is not present in the proximal tubule [17], at variance with the proximal tubule of the mouse, which expresses both NHERF1 and NHERF2 [18]. Heterozygous and homozygous disruption of the NHERF1 gene in mice has been shown to result in an increase in urinary phosphate excretion suggesting the inability of NHERF2 to compensate for NHERF1 defects. In humans NHERF2 is only weakly detected in the proximal tubule [19]. Disruption of NHERF2 gene in mice does not mimic NHERF1 gene disruption confirming that the functions of these two proteins are distinct [20].

Co-immunoprecipitation studies suggest that NPT2c may also interact with NHERF1-PDZ1 in rats. This interaction was not reproduced in renal cells in culture. NPT2c sequence does not contain PDZ binding motifs [21]. The mechanism of this interaction is unknown. We cannot exclude that the mutation described here could also alter NPT2c-NHERF1 interaction and NPT2c targeting.

These results, together with those of previous studies (9) indicate that mutations in the NHERF1 gene can alter renal phosphate transport by various mechanisms according to the location of the mutation in the functional domains of the protein. The study reported here identifies a new mechanism of renal phosphate loss independent of PTH or FGF23 and establishes the role of the first PDZ domain of NHERF1 in controlling phosphate homeostasis.
